# Structural and Functional Change in Albino Rat Retina Induced by Various Visible Light Wavelengths

**DOI:** 10.3390/ijms23010309

**Published:** 2021-12-28

**Authors:** Sachiko Kaidzu, Tsutomu Okuno, Masaki Tanito, Akihiro Ohira

**Affiliations:** 1 Department of Ophthalmology, Faculty of Medicine, Shimane University, Izumo 693-8501, Shimane, Japan; okuno@j.email.ne.jp (T.O.); mtanito@med.shimane-u.ac.jp (M.T.); aohira@med.shimane-u.ac.jp (A.O.); 2 Occupational Ergonomics Research Group, National Institute of Occupational Safety and Health, Tama-ku, Kawasaki 214-8585, Kanagawa, Japan

**Keywords:** retinal degeneration, visible light, electroretinograms, outer nuclear layer

## Abstract

The effects of visible light, from short to long wavelengths, on the retina were investigated functionally and histologically. The left eyes of Sprague–Dawley albino rats (6-weeks old, *n* = 6 for each wavelength) were exposed to seven narrow-band wavelengths (central wavelengths, 421, 441, 459, 501, 541, 581, and 615 nm) with bandwidths of 16 to 29 nm (half bandwidth, ±8–14.5 nm) using a xenon lamp source with bandpass filters at the retinal radiant exposures of 340 and 680 J/cm^2^. The right unexposed eyes served as controls. Seven days after exposure, flash electroretinograms (ERGs) were recorded, and the outer nuclear layer (ONL) thickness was measured. Compared to the unexposed eyes, significant reductions in the a- and b-wave ERG amplitudes were seen in eyes exposed to 460-nm or shorter wavelengths of light. The ONL thickness near the optic nerve head also tended to decrease with exposure to shorter wavelengths. The decreased ERG amplitudes and ONL thicknesses were most prominent in eyes exposed to 420-nm light at both radiant exposures. When the wavelengths were the same, the higher the amount of radiant exposure and the stronger the damage. Compared to the unexposed eyes, the a- and b-waves did not decrease significantly in eyes exposed to 500-nm or longer wavelength light. The results indicate that the retinal damage induced by visible light observed in albino rats depends on the wavelength and energy level of the exposed light.

## 1. Introduction

Light is largely classified according to its wavelength: ultraviolet (UV) light has a wavelength of 400 nm or less, visible light has a wavelength of 400 to 750 nm, and infrared light has a wavelength of 750 nm or more. Since most of the visible light penetrates the cornea, lens, and vitreous body and reaches the retina, the effects of visible light on the retina have been investigated for a long time. The most well-known effect is the damage to retinal photoreceptor cells. The first report was by Noell et al. [1], who found visible light-damaged photoreceptor cells and that light damage can be classified into two types: class I (damage induced by low-intensity light exposure for long durations) and class II (damage induced by relatively high-intensity light exposure for short periods). Regarding the latter, the retinal damage increases in response to shorter wavelengths—that is, the high-photon-energy portion of the visible spectrum (400–500 nm). This damage, referred to as the “blue-light hazard,” has been observed in monkeys [2,3] and rats [4,5,6]. Recently, with the widespread use of tablets and smartphones, it has been suggested that the blue light from these electronic devices using light-emitting diodes (LEDs) may cause blue-light hazard [7]. In addition, as it has become clear that oxidative stress is involved in the pathogenesis of age-related macular degeneration (AMD), antioxidants, such as polyphenols, that are effective in inhibiting the onset or progression of AMD have been actively pursued [8,9]. In these studies, animal models of light-induced retinal damage are often used, especially rats and mice, which are widely used because they are easy to maintain and treat. Using this rodent model, the exposure conditions (wavelengths, exposure times, and light sources) have varied among researchers [10]. The easiest and most common model is one in which the animals are maintained freely under white fluorescent light. However, white fluorescent lamps have several wavelength peaks between 400 and 650 nm. Therefore, it is unknown which wavelengths were involved in the retinal damage caused by irradiation and to what extent. Furthermore, it is difficult to accurately ascertain the amount of light exposure in the eye because animals move freely and sleep.

Unlike visible light with short wavelengths that cause blue-light hazards, the effects of visible light with long wavelength on the retina have not been well studied except for near-infrared light because it is thought that it is extremely hard for visible light with long wavelength to cause injury to the retina [11]. Near-infrared light at 670 nm, used for photobiomoduoation, has been reported to have retinal protective effects in animal models of retinal diseases, such as AMD [12], diabetic retinopathy [13], and retinopathy of prematurity [14]. It has also been reported that irradiation of near-infrared light at 670 nm improves the decline in retinal function due to aging [15]. However, little research has been done on the effects of other long-wavelength visible light on the structure and visual function of the retina.

In the current study, we measured the exposure dose exactly and exposed the rat retinas to seven different wavelengths included in white fluorescent lamps (420, 440, 460, 540, 580, 615 nm) and maximum absorption wavelength of rhodopsin (500 nm) with narrow bandwidths and two different doses. We evaluated the effects of visible light on the retina, particularly the injury to the retina of short-wavelength visible light below 500 nm, and the effects on the visual function and structure of the retina of long-wavelength visible light above 540 nm. There is no other report on the effects of visible light of a wide range of wavelengths, from short to long, on the retina, where the wavelengths and energy levels are precisely set and exposed.

## 2. Results

### 2.1. ERG

**Figure 1:**
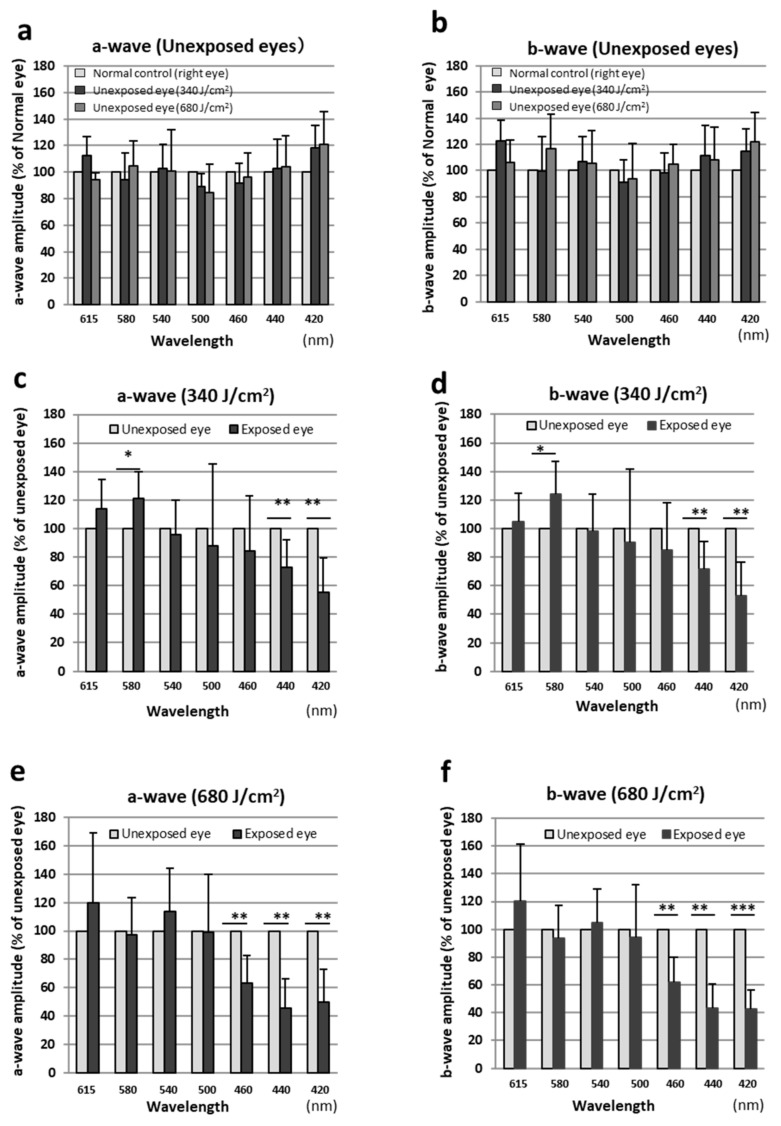
Measurement of retinal function by electroretinography. Data were expressed as mean ± SD. (**a**) The a-wave amplitude (% of the amplitude in normal control right eyes) for unexposed right eyes. (**b**) The b-wave amplitude (% of the amplitude in normal control right eyes) for unexposed right eyes. (**c**) The a-wave amplitude (% of the amplitude in unexposed right eyes) for eyes exposed to 340 J/cm^2^ light. (**d**) The b-wave amplitude (% of the amplitude in unexposed right eyes) for eyes exposed to 340 J/cm^2^ light. (**e**) The a-wave amplitude (% of the amplitude in unexposed right eyes) for eyes exposed to 680 J/cm^2^ light. (**f**) The b-wave amplitude (% of the amplitude in the unexposed right eyes) for eyes exposed to 680 J/cm^2^ light. Both eyes were compared using the paired *t*-test, and between-group comparisons were performed using the unpaired *t*-test. * *p* < 0.05, ** *p* < 0.01, *** *p* < 0.001. *n* = 6 in each group.

Retinal function was determined by ERGs. Figure 1 shows the amplitudes of the a- and b-waves from rats seven days after exposure and normal control rats. We compared the unexposed right eye (Exposure−/Anesthesia+) with the normal control right eye (Exposure−/Anesthesia−) to confirm if it was affected by the exposure and anesthesia. The amplitude of the right eye of the normal control was set as 100%, and the amplitude of each eye was quantified. Since there was no significant difference between the a- and b-wave compared to the normal control (Figure 1a,b), the amplitude of the unexposed (right) eye was set at 100% and compared to the amplitude of the exposed (left) eye. The ERGs obtained from rats exposed to 340 J/cm^2^ light indicated that 73% and 55% of the a-wave amplitudes were statistically significantly lost in eyes exposed to 420- and 440-nm light, respectively (Figure 1c). Statistically significant loss of the b-wave amplitude also occurred in the eyes exposed to 440-nm (72%) and 420-nm (53%) light (Figure 1d). On the other hand, the amplitude was statistically significantly increased in the retina irradiated with 340 J/cm^2^ of 580-nm light, with a 21% increase in the a-wave and a 24% increase in the b-wave (Figure 1c,d). The ERGs obtained from rats exposed to 680 J/cm^2^ light showed statistically significant decreases in the a- and b-wave amplitudes in eyes exposed to wavelength light shorter than 460 nm (Figure 1e,f). The a- and b-wave amplitudes in the eyes exposed to 460-nm light decreased by 63% and 62%, respectively. In the eyes exposed to 440-nm light, 45% and 42%, respectively, of the a- and b-wave amplitudes were lost, and in the eyes exposed to 420-nm light, the respective losses were 50% and 42%.

### 2.2. Morphologic Evaluation by Quantitative Histology

**Figure 2:**
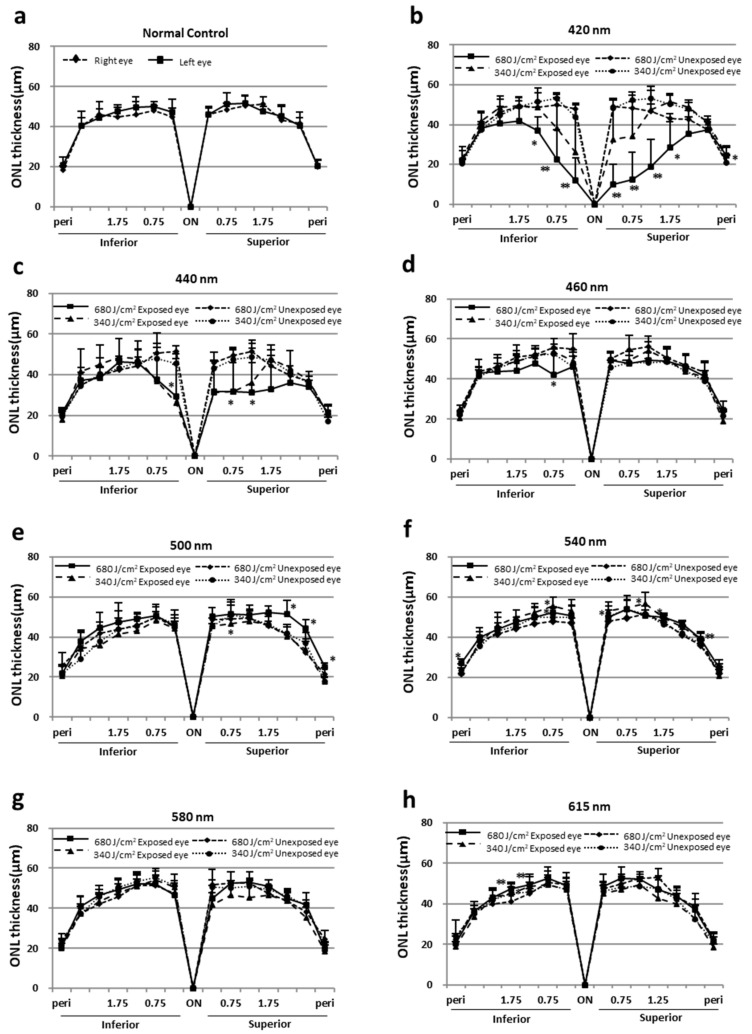
ONL thicknesses in normal controls and those exposed to light. (**a**) ONL thickness of normal control retinas. (**b**–**h**) ONL thicknesses of light-exposed retinas (left eye) are compared with those of unexposed retinas (right eye) at the following wavelengths: 420 nm, 440 nm, 460 nm, 500 nm, 540 nm, 580 nm, and 615 nm. The data are expressed as the mean ± SD (*n* = 6 in each group). Comparisons between groups were performed using the unpaired *t*-test. * *p* < 0.05 and ** *p* < 0.01 vs. unexposed eyes.

We assessed the retinal damage using morphologic methods with H & E staining. There was no difference in heat between the left and right retinas of the normal controls (Figure 2a). Thinning of the ONL occurred in the retinas exposed to visible light of wavelengths below 460 nm (Figure 2b–d). However, the thinning was not marked for 460-nm light (Figure 2d). In the retinas exposed to 440- and 420-nm light, marked decreases in the ONL thickness occurred in the inferior and superior hemispheres, and damage induced by 340 J/cm^2^ light was milder than that induced by 680 J/cm2 light (Figure 2b,c). Although the retinal damage was most severe around the optic nerve head, damage was more severe in the superior retina compared to the inferior retina. On the other hand, no thinning of the ONL was observed in the retinas exposed to light of wavelengths above 500 nm (Figure 2f,g), and the retinas irradiated with 500-, 540-, and 615-nm light were partially thicker than the unexposed retina (Figure 2e,f,h).

**Figure 3:**
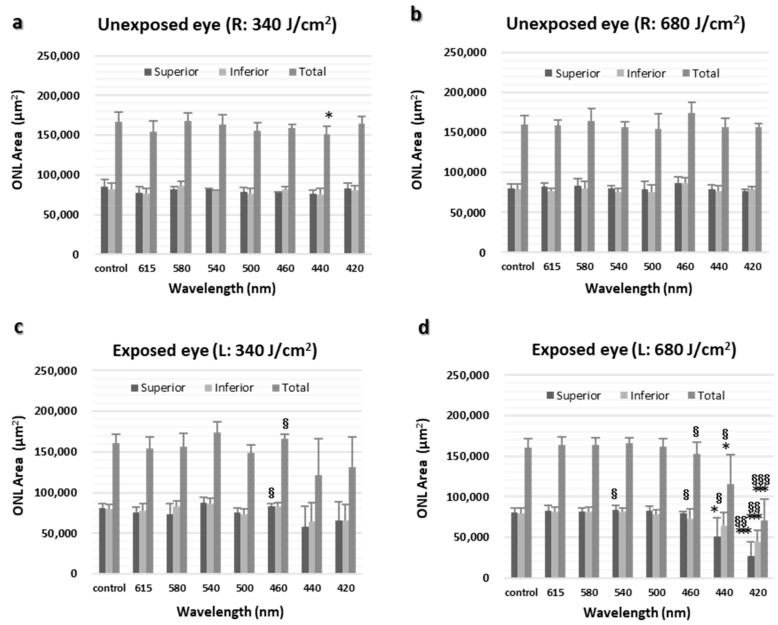
ONL area. (**a**) ONL area of normal retina (right eye) and opposite retina exposed to 340 J/cm^2^ (right eye). (**b**) ONL area of normal retina (right eye) and opposite retina exposed to 680 J/cm^2^ (right eye). (**c**) ONL area of normal retina (left eye) and retina exposed to 340 J/cm^2^ (left eye). (**d**) ONL area of normal retina (left eye) and retina exposed to 680 J/cm^2^ (left eye). L, left eye; R, right eye. The data are expressed as mean ± SD (*n* = 6 in each group). Both eyes were compared using the paired *t*-test, and between-group comparisons were performed using the unpaired *t*-test. * *p* < 0.05 and *** *p* < 0.001 vs. normal control eyes and ^§^ *p* < 0.05, ^§§^ *p* < 0.01, and ^§§§^ *p* < 0.001 vs. unexposed eyes.

The ONL area in Figure 2 was calculated separately for superior and inferior area up to 1.75 nm from the optic nerve head, where light-induced thinning of the ONL was pronounced (Figure 3a–d). ONL thinning was observed in retinas exposed to 680 J/cm^2^ light at wavelengths of 460 nm or less and was particularly pronounced in retinas exposed to light at 440 and 420 nm. In addition, ONL thinning was more pronounced in the superior retina compared with the inferior retina (Figure 3d).

**Figure 4:**
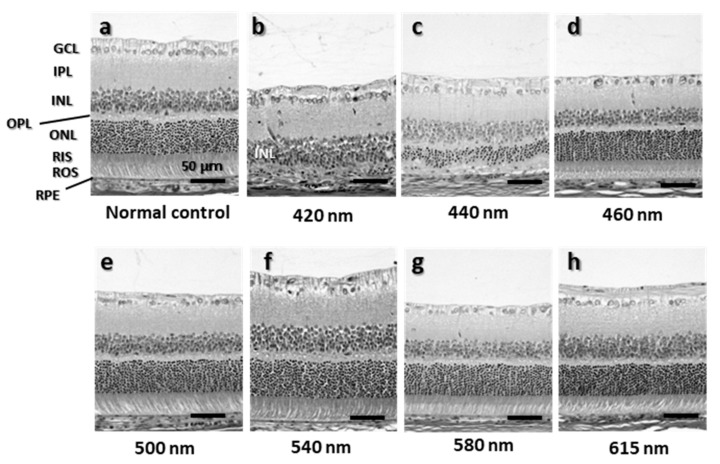
H&E of the retina 500–1000 µm superior the optic nerve. (**a**) Normal control retinas. (**b**–**h**) Light-exposed retinas exposed to following wavelengths: 420 nm, 440 nm, 460 nm, 500 nm, 540 nm, 580 nm, and 615 nm. GCL, ganglion cell layer; IPL, inner plexiform layer; INL, inner nuclear layer; OPL, outer plexiform layer; ONL, outer nuclear layer; RIS, rod inner segments; ROS, rod outer segments; RPE, retinal pigment epithelium. Bar = 50 µm.

The structure of the retina 500–1000 μm superior to the optic nerve of the eye exposed to 680 J/cm^2^, where light-induced thinning of the ONL by short wavelength light was most pronounced, was observed (Figure 4). The most severe damage was observed in the retina exposed to 420-nm light, where the ONL, rod inner segments (RIS), and rod outer segments (ROS) almost completely disappeared (Figure 4b). In retinas exposed to 440-nm light, the ONL remained but was thinning to about half the thickness of normal controls, RIS and ROS arrangements were disordered, and the interface was unclear (Figure 4c). In the retinas exposed to other wavelengths of light, ONL thinning was not observed, and the retinas were almost intact compared with normal control retinas (Figure 4a,d–h).

## 3. Discussion

In the current study, we measured the exposure dose precisely and exposed the rat retinas to seven different wavelengths, from 420 to 620 nm, in two different doses. Extensive damage occurred in eyes exposed to short wavelengths, with light less than 440 nm being particularly hazardous, and when using the same wavelength, a long exposure time caused more severe damage. No retinal damage caused by visible light at greater than 500-nm wavelength was observed.

It has already known that short-wavelength blue light was highly damaging to retina and that the efficiency of induction of damage rapidly increases below 500 nm with decrease in the wavelength [3,6]. The same result was obtained in this study, indicating that most of the retinal damage caused by white fluorescent light comes from this short-wavelength visible light (i.e., blue light hazard). In addition, although there were only two levels of radiant exposure in this study, it was also shown that the higher the amount of radiant exposure, the stronger the retinal damage. Currently, it is difficult to compare the damage to the retina caused by different light sources or different wavelengths because there is no adequate index. However, if it is possible to calculate the ED^50^ (the amount of energy that is half the value of the normal control) of ERG amplitudes and ONL thickness by exposure to more different amounts of energy, it may be possible to compare retinal damage with different light sources or different wavelengths.

In this study, we investigated the effect of 500 nm, the maximum absorption wavelength of rhodopsin, on the retina in addition to six peaks in white fluorescent light because light-induced retinal damage in rats is rhodopsin-mediated [1,16,17,18]. In knockout mice lacking rhodopsin in the photoreceptors, transcription factor AP-1, a central element in the apoptotic response to light [9,19,20], is not activated, resulting in completely protecting against light-induced apoptosis [17]. Therefore, rhodopsin is essential for generating or transducing the intracellular death signal induced by light. However, in the current study, 500-nm light, almost the absorption peak of rhodopsin, did not induce damage in the rat retinas, which agreed with previous reports that green light is less harmful than blue light [10,18]. Gorgels and van Norren studied the specific exposure conditions for funduscopic threshold damage and reported that the mean funduscopic threshold doses were 1145 ± 332 J/cm^2^ in 500-nm light and 1612 ± 158 J/cm^2^ in 550-nm wavelength light [6]. Regarding the threshold dose in 600-nm light, although the investigators used the maximal available dose of 3235 J/cm^2^, the retinal damage was sub-threshold. There were some differences between animal species (pigmented and albino rats) and age; however, compared with these values, the radiant exposures used in the current study, 680 and 340 J/cm^2^, might be too low to induce light damage. Indeed, previous studies have reported rat retinal damage induced by green light [1,5,18,21]. However, the radiant exposures in those studies also were low compared with the value reported by Gorgels and van Norren [6]. This difference might have resulted from the spectral distribution of light used in the experiments. In previous studies, the green light had relatively wide bandwidths (70 nm) and included blue light (465 nm) [5,20]. We observed retinal damage induced by 460-nm blue light in a recent study. Thus, short wavelength light included in the exposed light might have induced light damage in the retina. Understandably, the current results do not necessarily suggest that light with wavelength longer than 500 nm is safe for the retina. Krigel et al. reported that green LED light exposure for one month induced retinal damage in albino rats [22]; therefore, the possibility of long-term irradiation causing retinal injury cannot be excluded. Further studies are needed to determine if green light is less harmful.

Interestingly, the amplitudes of the a- and b-wave of ERG increased, and the ONL became thicker in the retina exposed to longer wavelengths than 540 nm. Since there are few reports on the effects of long-wavelength visible light on the retina, it is difficult to speculate the cause of these changes, but near-infrared light at 670 nm may provide a possible clue. Visible light at 670 nm not only has been shown to have retinal protective effects in animal models of retinal disease [12,13,14] but also improves age-related retinal function [15]. Sivapathasuntharam C. et al. reported that exposure of 7- and 12-month-old mice with 670-nm near-infrared light for 15 min daily over one month improved the ERG amplitude decline in both a- and b-waves and speculated that this may be due to the additional adenosine triphosphate production for photoreceptor ion pumps and reduction in aged inflammation [15]. Although it is not possible to make simple comparisons because of the differences in animals and exposure conditions as well as wavelengths, it may be that visible light around 600 nm has the same effect on improving visual function as near-infrared light at 670 nm. However, these are just guesses, and further research is needed.

The severity of retinal light damage differs markedly by ocular region and has been the topic of several studies since Rapp and Williams [23] first reported such differences. Along the vertical ocular meridian, the superior retina is damaged more severely than the inferior retina, and the central retina is damaged more severely than the peripheral retina [5,24,25,26]. Tanito et al. studied the extent of retinal light damage in all retinal regions and reported that the ONL thinning was greatest at 1 to 1.5 mm superior and superotemporal to the optic nerve head and that most damage was in the superotemporal region of the fundus [27]. The reason for the regional differences in retinal damage remains unclear. Several factors have been suggested as potential candidates for causing susceptibility to retinal damage leading to the regional differences, e.g., rhodopsin content [28]; fatty acid composition [29]; cytoprotective molecules, such as growth factors/cytokines/neurotrophins [30,31,32]; antioxidants [33]; and chaperones, such as heat shock proteins [32]. The regional differences of those factors in the retina might reflect the regional differences in the retinal damage. In the current study, the ONL thickness measured along the vertical ocular meridian represents an almost symmetric reduction curve for the superior and inferior retina. We suspect that the angles at which light approaches the inside of the eye might be important factors inducing this difference because in the current study, light was presented at a right angle to the corneal center, which means that light entered the eye approximately parallel to the optical axis. Stone et al. also reported that when the room light in the animal facility was changed from the ceiling to the side wall, light damage in the superior ocular hemisphere was largely prevented [32], indicating that the light angle can affect the area in which the damage caused by intense light occurred. Nevertheless, the upper and lower retinal injuries were not completely equal, and the inferior retina incurred slightly less severe damage. The distribution of various factors in the retina, as described above, may have affected the degree of light-induced retinal injury.

The time of the onset of the light exposure is important because the susceptibility to light damage is affected by circadian rhythm [34,35], with the susceptibility peaking during darkness. In the current study, we exposed the rats to light during daytime (7:00 AM–7:00 PM), which prevented the start of the exposure time from overlapping nighttime and daytime. Comparing each group, it is thought that the effect of the circadian rhythm is almost offset.

In studies of the central and peripheral nervous system, ketamine/xylazine is recommended and used widely. We used ketamine/xylazine to induce anesthesia in rats so that we calculated and exposed the light with accurate dose to the retina. Halothane, an inhaled anesthetic agent, protects against photoreceptor apoptosis in light-induced retinal damage models [36]. Pretreatment with ketamine/xylazine anesthesia recently was reported to protect retinas against light damage, thus reducing photoreceptor cellular death [37]. Rats treated with ketamine/xylazine for 1 h followed by a 2-h recovery phase before light exposure had decreased retinal degeneration. Because we continuously administered the anesthetics (i.e., without a recovery phase), the current study did not determine if the protective effect of ketamine/xylazine affected the results. However, the exposure times varied based on the wavelength (Table 1) (44.5 min with 581-nm light and 399.0 min in 615-nm light); therefore, the anesthetic doses also varied. We cannot deny the possibility that the anesthesia affected the results.

It is important to note that, as in many other studies [10], the animals used in this study were albino rats, as they are more susceptible to light damage to the retina. Pigmented rats are more resistant to light, and they need to be exposed to intense light for several days (5–7 days) to cause retinal damages [38,39,40], while albino rats need less than 24 h of light exposure to cause retinal damages. There is a possibility that retinal damage may not occur in pigmented rats even if they are exposed to the same light conditions that caused retinal damage in this study, and therefore, the results of this study may not be directly applied to pigmented rats or other pigmented animals. The exact amount of light energy required to cause damage to the retina of pigmented rats is not known, and further study is needed.

In this study, we measured the exposure dose exactly and exposed the rat retinas to seven different wavelengths included in white fluorescent lamps (420, 440, 460, 540, 580, 615 nm) and maximum absorption wavelength of rhodopsin (500 nm) with narrow bandwidths and two different doses and evaluated the effects of visible light on the retina. Most of the retinal damage induced by white fluorescent light exposure was due to light with a wavelength of 460 nm or less (i.e., blue light hazard). Rodent models of acute and intense light damage can be useful to test the effects of blue-light hazard reported in humans and monkeys. On the other hand, no retinal damage caused by visible light of greater than 500-nm wavelength was observed. Our result showed that the retinal damage induced by visible light observed in albino rats depended on the wavelength and energy level of the exposed light.

## 4. Materials and Methods

### 4.1. Animals

All procedures were performed according to the ARVO Statement for the Use of Animals in Ophthalmic and Vision Research and The Shimane University Guidelines for Animals in Research (IZ29-76). Male Sprague–Dawley 5-week-old rats were obtained from Charles River Laboratories Japan Inc. (Kanagawa, Japan) and maintained in our colony room for 7 to 10 days before the experiments. The light illuminance on the cage floor was 10 to 20 lux. All rats were kept in a 12-h (7 AM to 7 PM) light–dark cycle.

### 4.2. Light Exposure

Before light exposure, the rats were kept in dark boxes. After anesthesia was induced by an intramuscular injection of a mixture of ketamine (120 mg/kg) and xylazine (6 mg/kg), the pupils were dilated with 0.5% tropicamide and 0.5% phenylephrine hydrochloride eye drops (Santen Pharmaceuticals Co., Ltd., Osaka, Japan). The left eyes were exposed to light, and the right eyes were not and served as controls. Rats were exposed to seven narrow-band wavelengths (central wavelengths of 421, 441, 459, 501, 541, 581, and 615 nm) with 16 to 29 nm in full-width-half-maximal bandwidth (Table 1) using a xenon.

**Figure 5:**
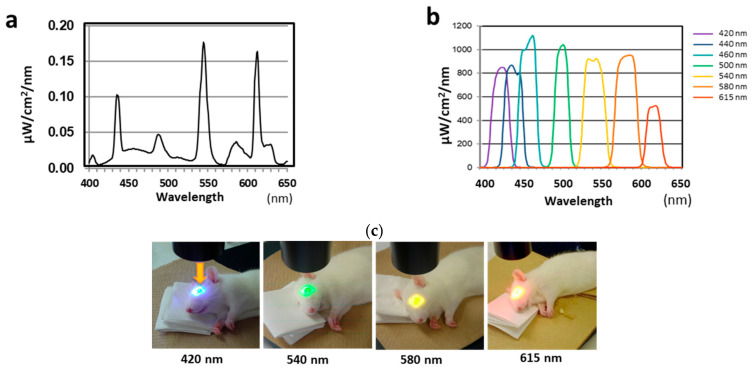
Spectral distributions of white fluorescent light from a xenon lamp and rats exposed to light. (**a**) Spectral distribution of white fluorescent light. (**b**) Spectral distribution of light from a xenon lamp source. (**c**) Rats exposed to light of different wavelengths. Arrow indicates the direction of the exposed visible light.

Lamp light source with bandpass filters (Asahi Spectra Co., Ltd., Tokyo, Japan) were used for estimated periods as described below. Seven peaks were chosen to correspond approximately to the six peaks of white fluorescent light (Figure 5a) and absorption peak of rhodopsin (498 nm) (Figure 5b). The light was presented at a right angle to the corneal center (Figure 5c). During exposure, diluted saline (×2) was instilled onto the corneal surface to prevent drying, and the other eye was closed with medical tape to prevent exposure to visible light. An intramuscular injection of an anesthetic drug was added to maintain the anesthesia. During exposure, the rats were placed on a body warmer and paper towels to prevent their body temperature from lowering. The rat body was also covered with a gauze towel. After exposure, the rats were kept under cyclic light (10–20 lux, 12-h light-dark–cycle) for 7 d until the electroretinogram (ERG) recordings and enucleation.

### 4.3. Determination of Duration of Light Exposure

**Figure 6:**
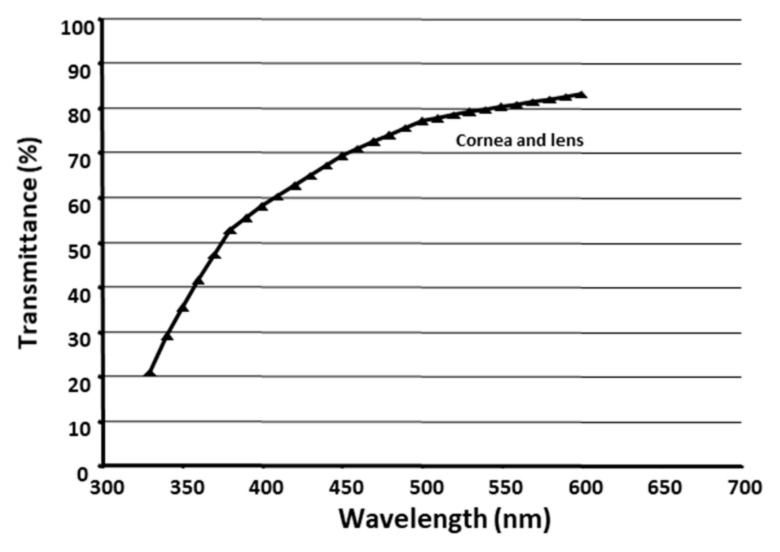
Spectral transmittance of the rat corneas and lenses. Values calculated from the transmittance of the corneas [41] and lenses [42].

Before the start of each exposure, the irradiance was measured using a radiometer (IL 1400A, International Light Technologies, Peabody, MA) connected to a silicon-photodiode detector (SEL033, International Light Technologies, Peabody, MA, USA) at the corneal position; the exposure duration was determined by dividing the target retinal radiant exposure (i.e., 340 or 680 J/cm^2^) by the measured irradiance and combined transmittance of the rat cornea [41] and lens [42] (Figure 6). The radiometer was calibrated before each light exposure. After the light exposure, the corneal irradiance was measured again and the average of the two measurements obtained, and the retinal radiant exposure was re-calculated to clarify that the retinal radiant exposure was around 340 or 680 J/cm^2^. Since the light source degraded with irradiation, the duration of light exposure increased correspondingly. The duration of light exposure was adjusted so that the retinal radiant exposure remained constant.

### 4.4. ERG

Seven days after the light exposure, flash ERGs were recorded (LS-W, Mayo Corporation, Aichi, Japan) according to previous reports [8,9]. Twenty minutes before the recording, the animals were anesthetized and the pupils dilated using the same methods as used for the light exposure. An LED electrode (Mayo Corporation, Aichi, Japan) was placed on both eyes. An identical reference electrode was placed in the mouth, and the ground electrode was placed on the left footpad. A single flash of light (10,000 cd/mm^2^, 5 ms) from the LED served as the light stimulation. The a- and b-wave amplitudes from the light-exposed and unexposed left and right eyes, respectively, were measured for statistical analysis. Both eyes from light unexposed and un-anesthetized animals served as normal controls

### 4.5. Histology

Both eyes were enucleated after the animals were killed by an overdose of anesthesia and then by cervical dislocation. The enucleated eyes were fixed in 4% paraformaldehyde (FUJIFILM Wako Chemical Corporation, Kanagawa, Japan) containing 20% isopropanol (FUJIFILM Wako Chemical Corporation, Kanagawa, Japan), 2% trichloroacetic acid (FUJIFILM Wako Chemical Corporation, Kanagawa, Japan), and 2% zinc chloride (FUJIFILM Wako Chemical Corporation, Kanagawa, Japan) for 24 h at room temperature. After alcohol dehydration, the eyes were embedded in paraffin, and 4-μm-thick sagittal sections containing the entire retina including the optic disc were cut. The outer nuclear layer (ONL) thickness was measured as reported previously [8,9,43] with slight modification. The retinal sections were stained with hematoxylin-eosin (H & E). For each section, digitized images of the entire retina were captured using a digital imaging system (VB-G25, Keyence Corporation, Osaka, Japan) at ×4 magnification with 1360 × 1024 pixels. Using Image J 1.32 software (National Institute of Health, Bethesda, MD), the ONL thicknesses were measured at 0.25, 0.75, 1.25, 1.75, 2.25, and 2.75 mm superior and inferior to the optic nerve head and at the periphery 100 μm from the inferior and superior edge of the retina. The ONL area was calculated separately for superior and inferior area up to 1.75 nm from the optic nerve head.

### 4.6. Statistical Analysis

The data are expressed as the means ± standard deviations (SDs). Comparisons of both eyes were performed using the paired *t*-test, and between-group comparisons were performed using the unpaired *t*-test (StatMate V 5.01; ATMS Co., Ltd., Tokyo, Japan). *p* < 0.05 was considered statistically significant.

## Figures and Tables

**Table 1:** Summary of Exposure Conditions.

Central Wavelength (nm)	Maximum Half Bandwidth (nm)	Transmittance (Cornea + Lens) (%)	Measured Irradiance at Corneal Surface (mW/cm^2^)	Estimated Radiant Exposure at Corneal Surface (J/cm^2^)	Estimated Irradiance at Retinas (mW/cm^2^)	Exposure Time (minutes)	Estimated Radiant Exposure at Retinas (J/cm^2^)
421	8	0.626	56.0–67.8	1086 ± 8	35.0–42.4	265.0–321.0	680 ± 5
			51.0–59.0	542 ± 3	32.0–36.9	153.0–178.0	339 ± 2
441	12	0.671	84.6–123.3	1011 ± 4	56.8–82.8	137.0–198.0	678 ± 3
			75.6–82.5	506 ± 3	50.7–55.4	103.0–111.0	340 ± 2
459	11.5	0.708	114.7–120.5	963 ± 5	81.2–85.3	132.0–140.0	680 ± 4
			113.0–126.0	480 ± 3	80.0–89.6	63.5–70.5	340 ± 2
501	9	0.770	85.5–129.0	891 ± 39	65.9–99.3	120.0–168.0	680 ± 30
			77.0–108.9	443 ± 31	59.3–83.8	71.0–91.0	341 ± 24
541	14	0.796	59.1–60.5	863 ± 9	47.0–48.1	235.0–242.0	679 ± 7
			58.3–59.4	427 ± 5	46.4–47.2	119.0–123.0	340 ± 4
581	14.5	0.818	59.2–170.2	833 ± 9	48.4–139.2	80.0–234.0	682 ± 8
			58.7–155.3	416 ± 3	48.0–127.0	44.5–118	341 ± 3
615	9.5	0.841	34.1–57.4	810 ± 4	28.6–48.3	235.0–399.0	681 ± 3
			37.6–42.6	405 ± 5	31.6–35.9	162.0–180.0	341 ± 4

The radiant exposure data are expressed as the means ± SD (*n* = 6 in each group). For all wavelengths, the upper row shows conditions for visible light with an irradiance of 680 mJ/cm^2^, and the lower row shows conditions for visible light with an irradiance of 340 mJ/cm^2^.

## Data Availability

The data that support the results of this research are available from the corresponding author upon reasonable request.
